# Association between physician’s sexual orientation, gender and their emotional response when evaluating suicidal patients

**DOI:** 10.1192/j.eurpsy.2025.728

**Published:** 2025-08-26

**Authors:** R. T. Monteiro, G. I. Zanella, T. P. Comissoli, P. B. Bolzan, Y. A. Ferrão

**Affiliations:** 1 Universidade Federal de Ciências da Saúde de Porto Alegre (UFCSPA); 2 Serviço de Psiquiatria, Hospital Materno Infantil Presidente Vargas (HMIPV), Porto Alegre, Brazil; 3 Vrije Universiteit Amsterdam, Amsterdam, Netherlands

## Abstract

**Introduction:**

Despite several efforts and public awareness campaigns, suicide rates are still rising in some countries, being third leading cause of death among 15-29-years-old. Some populations are at higher risk for suicidal behavior, such as LGBTIQ+ people and physicians. We found no literature regarding a possible association between physicians emotional response to suicidal patients (countertransference), sociodemographic factors and physicians mental health.

**Objectives:**

Identify a possible association between sociodemographic factors, countertransference and Burnout Syndrome.

**Methods:**

An anonymous web-based survey was implemented through the software REDCap, collected by snowball sampling. Participants were volunteers and could withdraw at any time. The study was approved by the University Ethics Committee. The survey consisted of the Informed Consent Form (ICF), Sociodemographic Questionnaire, Rating Scale for Countertransference (RSCT), which evaluate the main emotional responses (approximation, indifference or rejection). Scales also included Burnout Assessment Tool (BAT) and the Interpersonal Reactivity Index of Davis. Other scales were included, but are being developed in other studies.

**Results:**

From the 210 respondents, 179 (85.2%) completed the query: 108 (60.3%) were female; 166 (92.7%) were self-declared white-colored skin; 139 (77.7%) had a sexual partner. The mean age was 37.22 (SD=12.33), 65(36.3%) were medical residents; 112 (62.6%) were already specialists, 54 (48.2%) of those, declared to be psychiatrists. Indifference varies according to sexual orientation (p = 0,002), even when controlling for burnout (p < 0,001). Bissexual physicians had lower indifference rates than the other groups (Heterosexual: N=104, M 1,52, SD 1,52, CV 0,998; Homosexual: N=15, M 2,60, CV 0,870; Bisexual: N=17, M 0,64, SD 0,20, CV 1,332). Females had higher approximation rates when compared to male (Male: N = 53; M 15,71; SD 5,77; Female: N = 90; M 17,94; SD 3,70; p = 0,006). There’s no difference between gender when evaluating indifference and rejection. Homosexual and bisexual physicians experience more burnout (Heterosexual: N = 118, M 49,94, SD 14,5. Homosexual: N = 16; M 60,0; SD 15,2. Bisexual: N = 18; M 59,6; SD 4,6). F (2) = 5,51; p = 0,05.

**Conclusions:**

We find preliminary evidence that sexual orientation could influence emotional response when evaluating suicidal patients. Women have higher approximation. Both results remain the same when controlling for Burnout syndrome.

**Disclosure of Interest:**

None Declared

